# Improving multi-sport event ticketing accounting information system design through implementing RFID and blockchain technologies within COVID-19 health protocols

**DOI:** 10.1016/j.heliyon.2021.e08167

**Published:** 2021-10-20

**Authors:** Aji Nugraha, Debby Ratna Daniel, Anak Agung Gde Satia Utama

**Affiliations:** Study Program of Accounting, Department of Accountancy, Faculty of Economics and Business, Universitas Airlangga, Campus B, Jalan Airlangga 4, Surabaya 60286, Indonesia

**Keywords:** Accounting information system, Blockchain, COVID-19, Multi-sport event, RFID, Ticketing

## Abstract

To run a multi-sport event, it is necessary to have a design of accounting information system for ticket sales that can run efficiently and can reduce opportunities of fraudulent acts. A case study during the 18^th^ Asian Games 2018 shows that there were problems of inadequate ticket sales facilities for prospective spectators due to vendor diversion to the frictional problems such as venues located in various regions and protection of spectator rights in accordance with the purchased tickets. Some cases found in the multi-sport event were false seats and fictitious spectators allowed entrance to some arenas they did not have the right to enter, although they have gone through verification measures using line-of-sight barcoding technology. Some cases were also found during the 18^th^ Asian Games 2018, in which there was a problem of inadequate ticket sales facilities for prospective spectators due to a change of vendor. There were also frictional problems on venues which are spread in various regions and regarding protection of spectators’ rights per their purchased tickets. Moreover, it is fundamental that we take concern in the current pandemic situation, all event organizers are obliged to consider implementing health protocols issued by the World Health Organization (WHO) for safety to break the chain of COVID-19 infection. This study is conducted to identify the core of those problems and offer a solution by implementing radio-frequency identification (RFID) and blockchain technology to optimize the services applied in the multi-sport event, especially during and post-pandemic. Ticketing effectiveness for spectators are also challenged by budgetary and eco-friendliness issues.

## 1 Introduction

**Figure 1:**
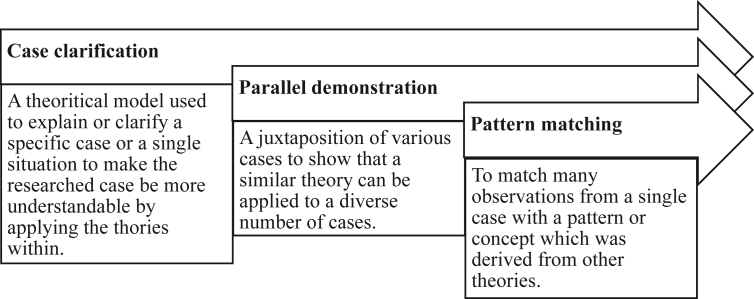
Analytical technique of this study in accordance with Neuman (2018).

**Figure 2:**
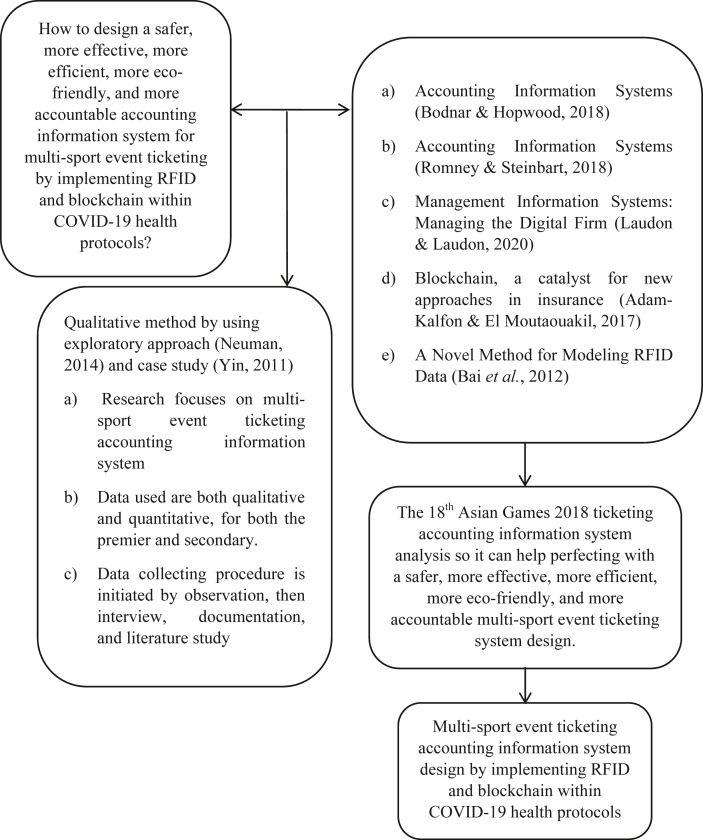
Conceptual framework of this study.

**Figure 3:**
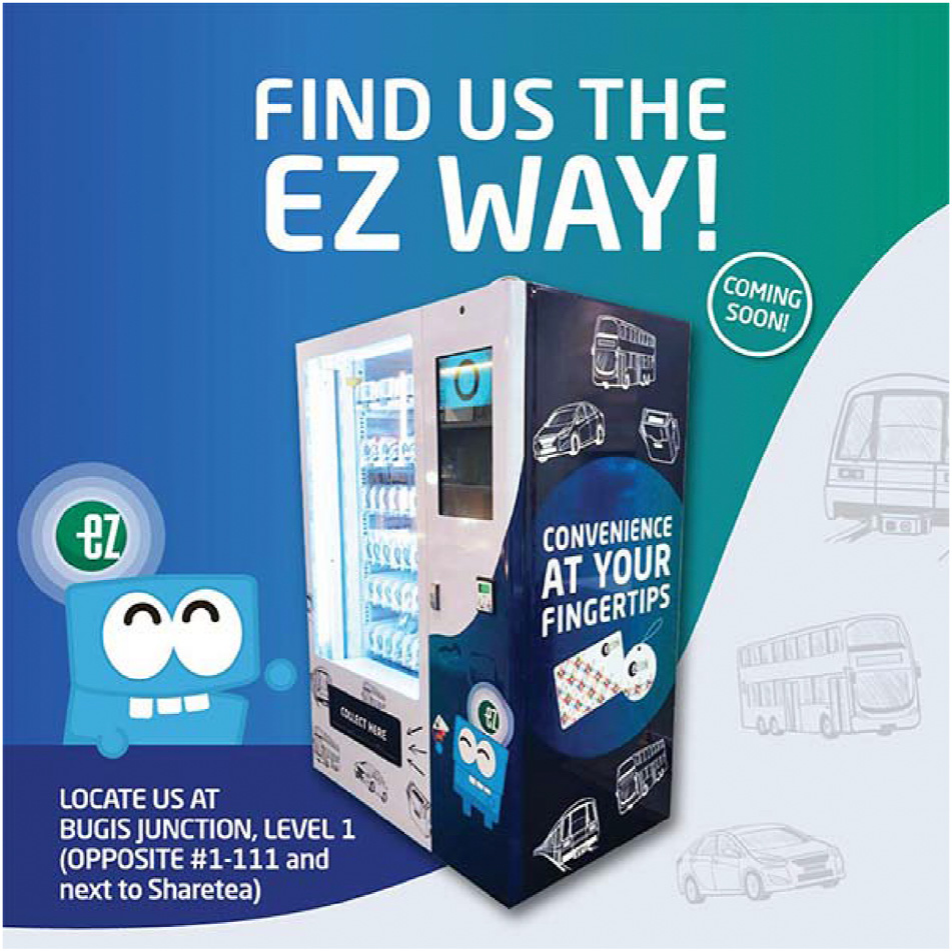
*EZ-Link Singapore*'s vending machine (*Kiosk Card Dispenser*^*+*^).

**Figure 4:**
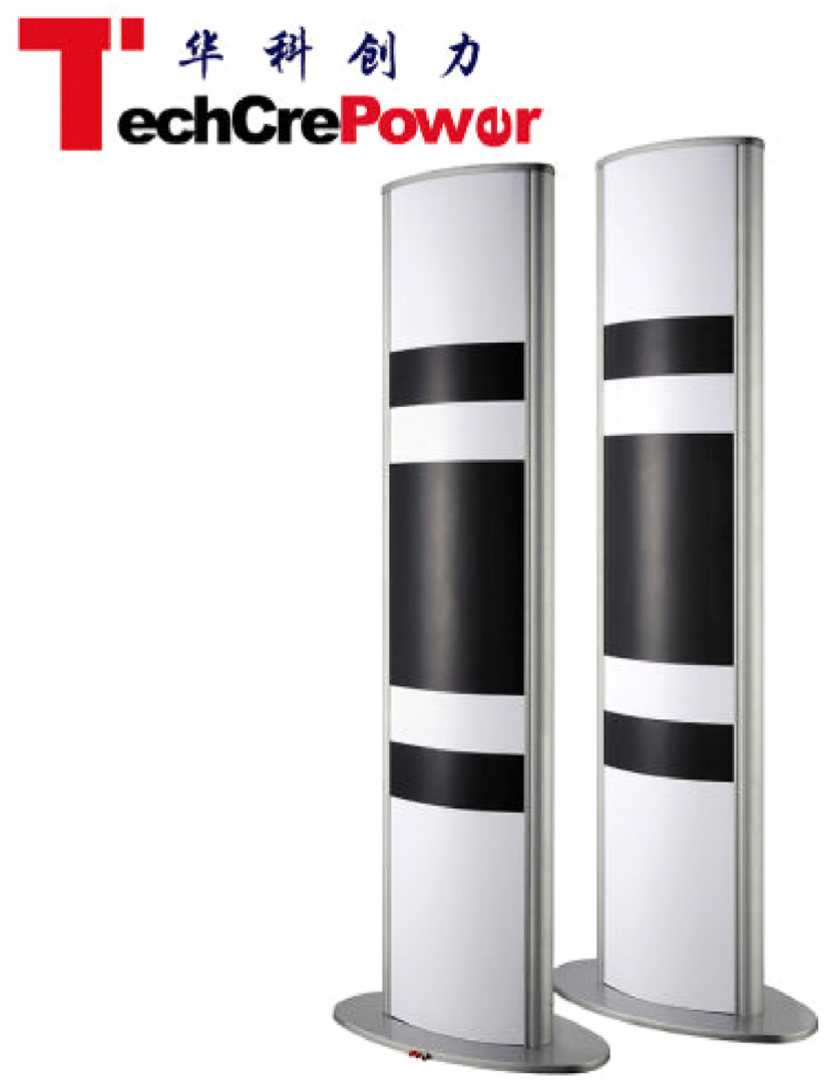
Reading range door access control UHF RFID gate reader.

**Figure 5:**
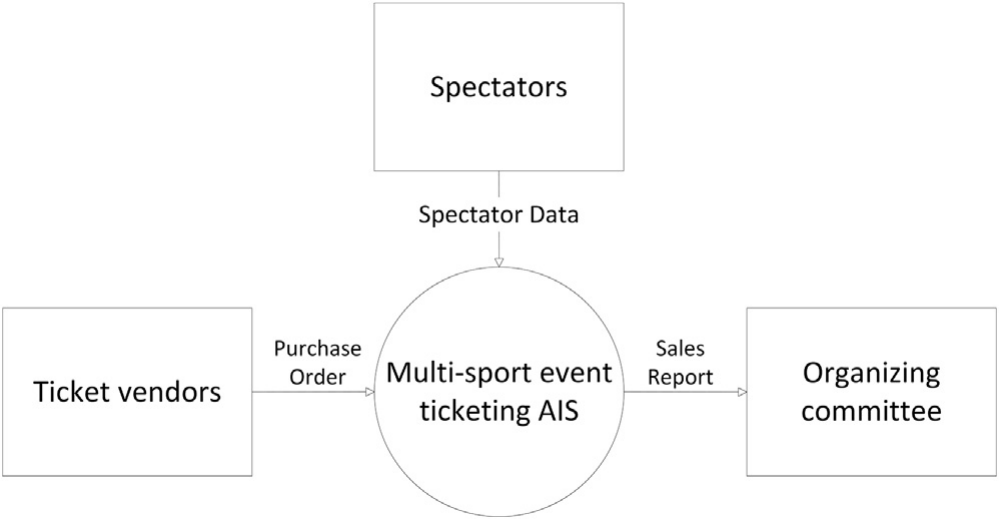
Context data-flow diagram (DFD) of the proposed accounting information system.

This proposed system may contribute in empirical studies to recommend a better ticketing system for either the event committee and third-party ticket vendors especially for multi-sport events so that it can run more safely, effectively, efficiently, eco-friendly, and accountable which can benefit to all parties involved by the implementation of radio-frequency identification (RFID) and blockchain technology in the system. These benefits may even make events, especially multi-sport events where there are many matches to choose with their various venues and schedules that can be disseminated by smart contracts and tags within the tickets. While in previous researches on this particular sector are often more focused on the ticketing system for public transportation and revenue management. This paper focuses more on the ticketing system for multi-sport events and other related events Figures 1, 2, 3, 4, and 5.

In this paper, there will be findings and discussions on how RFID and blockchain are two of the most sufficient technologies to be implemented in an accounting information system – especially in the field of ticketing management. While we already have various studies on these topics, we have not found a more focused study particularly regarding this issue. As to why this issue is relevant, is for us as a society to get prepared for upcoming multi-sport events and other events with similar ticketing architecture, either during or post-pandemic Tables 1 and 2.

**Table 1:** Total amount of international tourists in the 18^th^ Asian Games 2018.

No.	Country of Origin	People	Percentage
1.	China	10,375	12.97%
2.	Japan	10,038	12.55%
3.	South Korea	7,443	9.3%
4.	Malaysia	5,224	6.53%
5.	India	5,001	6.25%
6.	The Netherlands	2,341	2.92%
**Total**		40,422	50.52%
**Total Entirety**		80,000	100%

**Table 2:** Issues found around ticketing in the 18^th^ Asian Games 2018.

No.	Time	Arena	Issue
1.	Friday, 23 August 2018 Around 09:00 AM GMT+7	Senayan Tennis Indoor, Gelora Bung Karno Sports Complex, Jakarta	A Japanese journalist was found lost asking where to report the skateboarding match on that same day while it was held in another venue, specifically in Jakabaring Sport City, Palembang.
2.	Friday, 23 August 2018 Around 01:00 PM GMT+7	Senayan Tennis Indoor, Gelora Bung Karno Sports Complex, Jakarta	A couple of Thai spectators was allowed entrance by the barcode scanner while they do not even have any indoor volleyball tickets, for the match that was being held in Senayan Tennis Indoor at the time.

The implementation of advanced health measures, the use of RFID, and blockchain integrated with accounting information system may hopefully help resolving some of these issues. This technology will be used for a more accurate identification processing with the use of smart contracts which are unique to each user from athletes to committee members, or in this case – the live spectators.

Theories applied in this study are sales accounting information system, revenue cycle, agency theory, business process, pricing decisions, electronic data processing (EDP), RFID, blockchain, and artificial intelligence (AI). Some prior works are also put into use as references such as: a) Duran et al. (2014) which involves ticket bundling for sports and entertainment industry through optimizing revenue management to maximize event profitability by allocating relevant resources; and b) Chou et al. (2018) regarding resources including support systems related to services, security and time efficiency. These theories are then materialized through, for instance, disseminating classes in ticketing (Ellison et al., 2017).

RFID is implemented in various industries such as security, library, airline, and sports. It has proven to be a useful instrument that offers optimization of resources; customer service quality; accuracy improvement; and effective and efficient business processing through tagging with a unique unchangeable numbering system (Ahsan, 2015). Chen et al. (2014) stated that the usage of RFID is fundamental in this proposed system, as it can play the roles of tickets or ID cards, most RFID-tagged products are small and portable, and people may carry them in their daily life. Meanwhile Adam-Kalfon and El-Moutaouakil (2017) explained that blockchain technology can be used as a peer-to-peer (P2P) data lending as a transactional medium through the marriage of two concepts: asymmetric cryptography and distributed information technology architecture. Kim et al. (2019) identified that various users may provide new services and communicate to each other in the world of internet of things (IoT); which has more complexity than distributed IT architecture, which requires various hardware and software alike.

This study consists of six sections. The first section explains the background, gaps, and objectives of this paper, including the basis of selected research aspects and thoughts about ticketing accounting information system design through implementing RFID and blockchain technology and how they can contribute to making events more secure, effective, efficient, eco-friendly, and fraud-proof, so that this system will hopefully benefit all stakeholders involved. The second section dissects referenced literatures regarding relevant theories and hypotheses. The third section explains the methods being used to research related issues?? in a qualitative exploratory approach. The fourth section describes an analysis of the suggested system in this paper for a multi-sport event; how RFID and blockchain could ease and create a more accountable; multi-sport event; its data-flow diagram (DFD); entity relationship diagram (ERD); and relevant tables. The fifth section explains the study authors’ point of view regarding multi-sport events. Lastly, the sixth section concludes the paper as a whole, with a summary and recommendations for future studies.

## 2 Literature review

### 2.1 Agency theory

This research applies agency theory, which was popularized by Jensen and Meckling (1976), which introduces two entities into the proposed accounting information system: the principal and its agents. As anything related to accounting and sales has to be put through an auditing process, so the way to solve related issues is to use agency theory – as the theory itself is commonly used in researches in the field of auditing. As mentioned by Safriliana et al. (2018), in such architecture, we may encounter an information asymmetry, which mainly rests on the difference of information interests between agents and the principals. Using the Olympics as an example, the Olympics council as the principal overseeing the system, while Olympics committee and ticket vendors would act as agents.

### 2.2 Ticketing

The main issue of this research surrounds multi-sport event ticketing systems, which are not too reliable in general with some end user issues. To solve this issue, this research is based on papers such as Duran et al. (2014) that highlights ticketing system in both sports and entertainment industries, Chou, et al. (2018) regarding ticket price bundling, and Ellison et al. (2017) regarding online ticketing. Duran focuses on how revenue management can maximize profit by resource allocation such as flight seats, hotel rooms, and event tickets. These three measurements can also be implemented in multi-sport event ticketing. Which later can be functioned as an important point in creating strategic advantages and competitiveness in this emerging industry where this issue also involves appropriate pricing for spectator classes in a venue, when and to whom a discount can be applied, or which sports are suitable to be put into the portfolio. To support this idea, Chou researched *Taiwan High Speed Rail* (THSR), which prioritizes service, security, and timeliness. These particular characteristics can also be found in multi-sport events where spectators have the right to select their favorite matches and sports, similar to passenger classes in train ticketing. Therefore, a mapping system can be made to accommodate spectators to public transportation systems and getting involved in the social and economic impact on the multi-sport event. This issue is also found by Ellison regarding the use of Sydney's *Opal card* with its multimodal ticketing – namely, *MyMulti* which allows trips with various vehicles in a designated geographic area. Similar instances in multi-sport event are sports and matches to public transport selection for spectators in a smart, integrated systems.

### 2.3 Radio frequency identification (RFID)

The use of RFID is found in many sectors such as security, libraries, airlines, to sports. RFID has advantages to offer such as in optimizing resources, customer service, transaction accuracy, to business processes. Ahsan et al. (2011) emphasize that the system can be implemented by the use of RFID tags with unique numerical orders which cannot be changed for issues regarding security, which can also benefit spectators and multi-sport event organizers.

### 2.4 Blockchain

According to Adam-Kalfon and El Moutaouakil (2017), blockchain is a form of technology which can store data in a peer-to-peer (P2P) manner. This technology was born by the marriage of two concepts; the first one being asymmetric cryptography which allows engagements between public key and private systems, the second one is the information technology (IT) architecture which is distributed specialized in a P2P concept.

### 2.5 Internet of things (IoT)

Al-Fuqaha et al. (2015) believe that by the usage, the Internet of Things (IoT) is enabled by the latest developments in RFID, smart sensors, and Internet protocols; and by the definition, the IoT consists of a growing number of physical objects which are connected to the Internet at an unprecedented rate. In the world of IoT, various users can provide new services and interact to each other.

In a 2020 study, Tawalbeh, et al. refer IoT as a concept of connected objects and devices of all types over the Internet – either wired or wireless. In which organizations and individuals can communicate with each other remotely in a seamless connectivity, also called as hyperconnectivity. The term IoT was coined by Kevin Ashton in the year 1999 to promote the RFID concept – with sensors and actuators, which is also relevant in this paper.

Kim et al. (2019) found that an IoT which has more complex specifications than IT infrastructure is composed by many hardware and software. Similarly, blockchain is predicted to be applied in many Internet sectors, it can also be integrated into data, transaction, and authorization management. This issue is in line with many things needed by a multi-sport event organizer and committee to manage spectator data in a secure, effective, efficient, and fraud-proof infrastructure which can be traced from the very first ticket transaction, spectator data management can prove whether the ticket holder is allowed in the arena or not to spectator authentication by the ticketing department so the spectators can enter the arena on schedule, in their respective seat class and venue as stated in their purchased tickets.

## 3 Methods

A qualitative exploratory approach was used to conduct this research. This method was used to give certain information to other individuals and parties about the issues which are being researched, initiated by personal judgments of an issue based of historical data to then respond, gather, analyze, and interpret those data. After that, a test needs to be conducted to those issues, and then the general idea to be expanded and resumed by perfecting questions related to the research.

As for the method being used to conduct this research, a qualitative exploratory approach (Neuman, 2014) and a study case (Yin, 2016) are deemed as more appropriate to engage on such topics. These are considered regarding the research scope in the field of ticketing – especially on the accounting information system for multi-sport events, then both the primary and secondary data are either quantitative or qualitative, and the data collection procedure which was kickstarted by a first-hand observation, followed by interviews, and accompanied by documentations and literature review.

This research has been conducted in three steps. The first one is to determine variables and research objects, this analysis was based on an experience as a volunteer, particularly as a liaison officer to guests and spectators at the 18^th^ Asian Games 2018 in which were found some problems regarding proper service to spectators to security. The second one is to conduct interviews and documentations in which the interview was conducted with a partner AI company of the Indonesian Police at ensuring the safety of the 18^th^ Asian Games 2018, namely Nodeflux. Also, relevant interviews were conducted by asking relevant questions to spectators and volunteers alike for relevant issues regarding this research. Lastly, is to analyze problems and indicators the problems that were found need a set of indicators to make the research more focused on the main issue. The illustrative method as to conduct an analysis according to Neuman (2018, 490) is as follows, then followed by the conceptual framework:

## 4 Results and discussion

From some cases found in the 18^th^ Asian Games 2018 as a volunteer, all of those had spectators and journalists of foreign nationalities involved. With various countries of origin, backgrounds, and motives can be concluded as a frictional case. Therefore, an integrated ticketing accounting information system intertwined with visitor data as a whole - whichever their status - is needed.

Beyond those, the working security system was implemented by artificial intelligence (AI) induced face recognition system which was integrated into designated command centers in each regional police where the venues are located. But at the time it was limited to only process visitors of Indonesian nationality.

To get the multi-sport event ticketing system running, some processes regarding data flow are needed. There are three subjects involved to run this system, they are (1) to-be spectators as the buyers, (2) event organizing committee as the official ticket issuer, and last but not least (3) blockchain as the validator of the transaction. In the system there needs to be a planning for the matches which consists of (1) sports, (2) contingent, (3) region, (4) venue, (5) arena (with seats capacity), (6) schedule (including the date, hour, and match duration), and (7) seat class. After data collection, the main data will be shown in the ticketing system prepared for future purchases. The shown data will then direct to-be spectators to input the data to the buyers' account which are (1) full name, (2) home address, (3) email address, (4) phone number, and (5) special needs, such as the condition whether to bring children below 18 years old and adult spectators with disability concerns. Also, regarding the ongoing coronavirus disease 2019 (COVID-19) pandemic, it is necessary to include health measurements of the spectators such as body temperature and vaccination report. Payment will be transferred virtually via desired payment gateways whether it is bank account, virtual account (VA) or digital wallet. After payment checkout, a barcode will be sent to spectators' registered email. Multi-sport event organizers conduct a partnership with retail businesses as games partners to place kiosk card dispensers in the form of vending machines to dispense radio-frequency identification (RFID) tags attached ticket wristbands integrated with a near-field communication (NFC) technology connected with the blockchain records. The dispensers are modified with barcode scanners to scan the barcodes sent to each spectators’ email as shown on their smartphones.

In this proposed system, tickets will be integrated within the blockchain hierarchy. In this case, blockchain has a role as the transaction validator for tickets with verified purchase, so that the wristbands embedded with RFID tags do not have to be dispensed multiple times, spectators only have to walk pass the reading range door access UHF RFID gate reader which are available at the front gates of each venue and arena to scan the tags automatically. If the ticket is the same one as purchased, which are already stored in the blockchain hashes with smart contract specially made for transactions to allow the spectators to watch the match. If the ticket is not the same as purchased, then the RFID gate reader will ring an alarm with a beeping sound which will alarm the security staffs to direct them out form the arena. If then found another difficulty, then the issue will be directed to a designated command center in the venue or arena as needed, whether by handy talkie (HT) or face-to-face with the staffs.

Agency theory is implemented in this proposed accounting information system because this system has two types of entities. The first one acts as the business principal, mainly is the International Olympics Committee (IOC) to the designated Olympics council in the region (usually by continent, e.g., the Olympics Council of Asia (OCA) for Asia region) or just the IOC if it is the Olympics and Paralympics being held - by the hands of the multi-sport event committee to run the agreed system. Asymmetric information also happens between the IOC, the Olympics council and the multi-sport event committee so that the ticket sales is suitable with the seat capacity for spectators in the arenas for ticket vendors acting as agents who - as business entities will maximize their profit from ticket sales. This asymmetric information if occurs will be harmful for the spectators as consumers who have purchased the tickets as their desired matches, so an agency theory is needed so that their consumer rights can be distributed fair and square upon the relationship between the business principal and its agents.

In this proposed system there are several data-flows which are represented by some diagrams, generally called. As data-flow diagram (DFD). Those diagrams are mainly represented by a context DFD which is the contextual foundation of multi-sport event ticketing system. In the context DFD, the data flows from or to each other's entities. Those data are classified into three, the first one – namely, spectators for the spectator data, the second one being ticket vendors with purchase order which flows into the multi-sport event accounting information system (AIS), and out of the AIS for the sales report directed to the third one which is the organizing committee.

**Figure 6:**
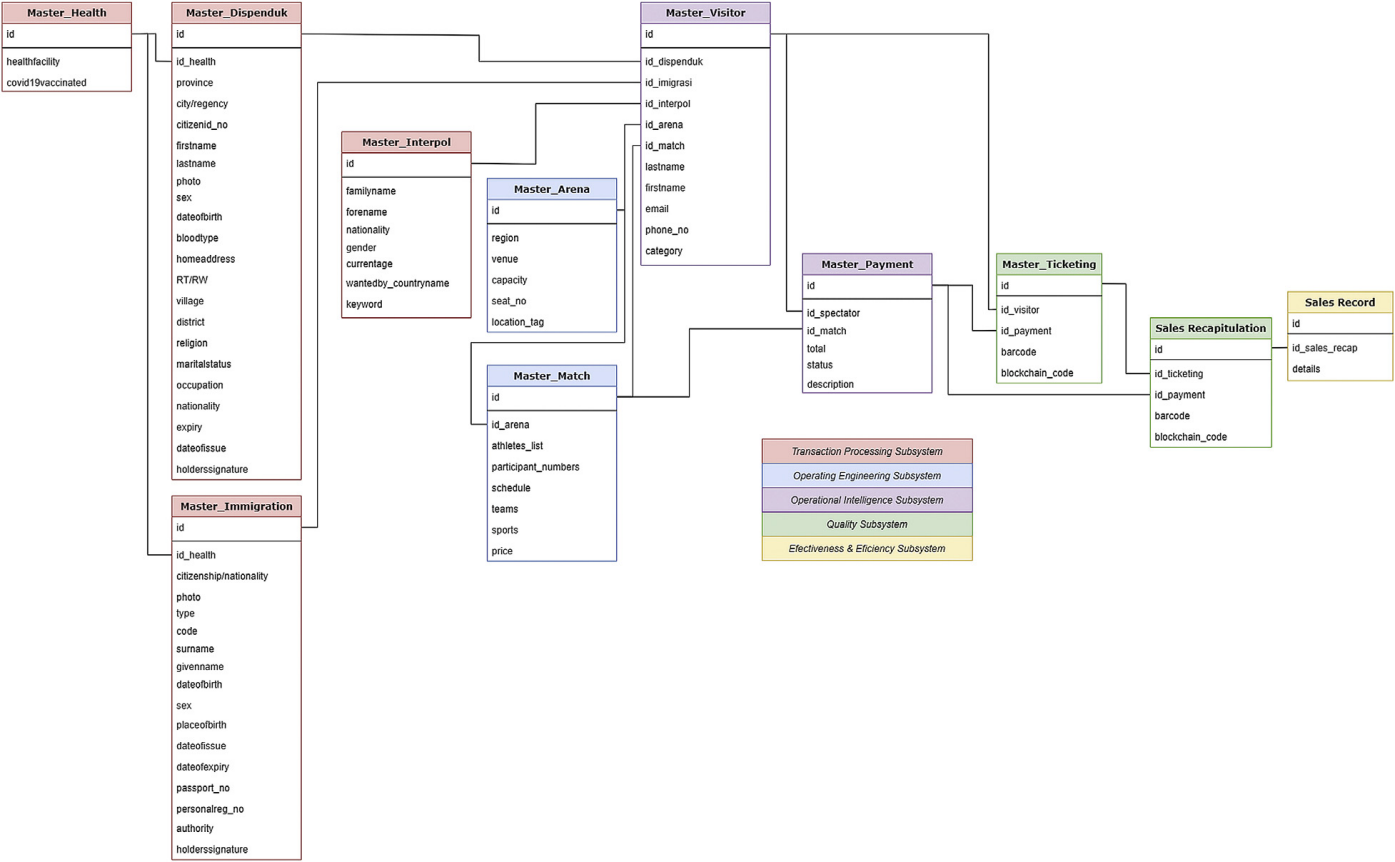
Entity relationship diagram (ERD) of the proposed accounting information system.

In Figure 6, we explore how each data is being transferred to each attribute to further the exchange of information. These data were then to be used as attributes for a working, effective, efficient, less-fraudulent, and sustainable ticketing accounting information system – especially for multi-sport events alike. With this, we explore the entity relationship diagram (ERD) used to build, develop, and maintain the accounting information system. There should be known that these attributes are dissected into five subsystems – they are specialized in transaction processing, operating engineering, operational intelligence, quality, and lastly in effectiveness and efficiency. These subsystems are essential to each of their specialties so that the data flow may work seamlessly within the system as a whole.

These diagrams – such as data-flow diagram (DFD) and ERD as aforementioned, are needed to counter some difficulties that may appear in the implementation of the new system. As there might be some weakness that can be found or even human error in the running of the system that may occur at any time, a controlling measure is needed to be applied for a more responsible and less-fraudulent workflow. This measure can be integrated into the sales record attribute as an accountability control.

As for the people responsible in running the system, there have to be two sides on the run. Those are the ones managing the system and the multi-sport event committee who run the games. In this scenario, the people might have a clearer vision as to what are the tasks that matches their job description and how can the system – as a control measure, be integrated into their jobs whether it is in or even after the games for accountability reasons.

The implementation of RFID and blockchain is agreed by some parties can benefit many sectors (Kuang and Su, 2013; Singh, 2020; Kim et al., 2019). However, some also disagree to apply these changes as for societal adaptation (Angell and Kietzmann, 2001) to budget issues (Hsu et al., 2020). These views are also apparent in interviews held with INASGOC officials and third-parties such as if there are still transactions being made and the tickets are being distributed fairly then there will be no need of such changes to apply new things like RFID and blockchain into the system. Flowcharts are also regarded as non-apparent in transaction systems.

## 5 Conclusions

With the implementation of RFID and blockchain in multi-sport event ticketing accounting information system design, a more efficient and accountable multi-sport event is expected. So, either the committee and the ticket vendors as partners of the event can benefit from this system as it can help them run the event way more easily at solving little issues that may cause a domino effect for the event's success and credibility in its stakeholders' eyes. Case-study centric papers are expected to be more structured so that the proposed system can be more beneficial at least as a reference to enhance a multi-sport event's safety, effectiveness, efficiency, eco-friendliness, and accountability. Also, more papers focusing on the use of RFID and blockchain in similar systems are encouraged for system's sustainability and further improvements in the future. While the system can also be applied to other events similar to a multi-sport event, such as amusement parks, exhibitions, and movie theaters. Application of artificial intelligence (AI) is also encouraged for future researches. Furthermore, this paper has some study limits, those are the effectiveness for spectators – especially, to scan the RFID tags, the eco-friendliness itself whether the ticket wristbands would be discharged or kept, and some budgetary issues.

## Declarations

### Author contribution statement

Aji Nugraha, Debby Ratna Daniel, Anak Agung Gde Satia Utama: Conceived and designed the experiments; ​Performed the experiments; ​Analyzed ​and interpreted the data; Contributed reagents, materials, analysis tools or data; Wrote the paper.

### Funding statement

This research did not receive any specific grant from funding agencies in the public, commercial, or not-for-profit sectors.

### Data availability statement

Data included in article.

### Declaration of interests statement

The authors declare no conflict of interest.

### Additional information

No additional information is available for this paper.
